# Physical Exercise and Brain Health: Exploring Activity Types to Enhance Cognition in Older Adults

**DOI:** 10.1093/geroni/igaf122.2748

**Published:** 2025-12-31

**Authors:** Anna Egbert, Isa-Marie Kreuzinger, Mark Brennan Ing

**Affiliations:** St. Joseph’s University New York, Brooklyn, New York, United States; St. Joseph’s University, Brooklyn, New York, United States; Hunter College, New York, New York, United States

## Abstract

With the aging population, identifying lifestyle factors that can promote cognitive resilience and sustain independence becomes increasingly important. Physical exercise is well-established as a key contributor to brain health across all age groups, yet further research is needed to determine the most effective exercise types for maximizing cognitive benefits in older adults. This study aimed to examine the relationship between perceived cognitive difficulties (assessed via the Patient Assessment of Own Functioning Inventory–PAOFI), various forms of physical activity (walking, light, moderate, strenuous, and strength/endurance exercises), and sedentary behavior in a sample of adults aged 60–80 years (n = 42). Hierarchical regression modeling revealed that demographic, socioeconomic, and health-related factors, along with physical activity, accounted for 87% of the variance in PAOFI total scores. Higher scores on PAOFI Memory Scale were linked to male sex (B=-4.591, p=.003, CI95%=-7.518, -1.665), Caucasian White race/ethnicity (B = 12.538, p=.006, CI95%=3.961, 21.116), and engaging in more physical activities of light (B = 2.982, p=.006, CI95%=.905, 5.058) and moderate intensity (B = 14.093, p=.004, CI95%=4.736, 23.451). Higher scores on PAOFI Higher Level Cognitive and Intellectual Functioning Scale were linked to less time spent on sedentary activities (B=-18.885, p=.002, CI95%=-30.450, -7.321) and more walking (B = 3.007, p=.008, CI95%=.837, 5.177). These findings highlight potential benefits of promoting movement-based interventions and provide a basis for developing targeted programs for older adults.

